# A Qualitative Study of Food Choice in Urban Coastal Esmeraldas, Ecuador

**DOI:** 10.1016/j.cdnut.2023.100093

**Published:** 2023-04-26

**Authors:** Jessica Uruchima, Cala Renehan, Nancy Castro, William Cevallos, Karen Levy, Joseph NS. Eisenberg, Gwenyth O. Lee

**Affiliations:** 1 Department of Epidemiology, School of Public Health, University of Michigan, Ann Arbor, MI, United States; 2 Carrera de Nutrición y Dietética, Universidad de San Francisco de Quito, Quito, Ecuador; 3 Centro de Biomedicina, Carrera de Medicina, Universidad Central, Quito, Ecuador; 4 Department of Environmental and Occupational Health, University of Washington, Seattle, WA, United States; 5 Rutgers Global Health Institute, Rutgers University, New Brunswick, NJ, United States

**Keywords:** Ecuador, urbanicity, food environment, food security, food safety, qualitative

## Abstract

**Background:**

Constraints on food choice increase risk of malnutrition worldwide. Residents of secondary cities within low- and middle-income countries are a population of particular concern because they often face high rates of food insecurity and multiple nutritional burdens. Within this context, effective and equitable interventions to support healthy diets must be based on an understanding of the lived experience of individuals and their interactions with the food environment.

**Objectives:**

The primary objectives of this study were to describe considerations that drive household decision making around food choice in the city of Esmeraldas, Ecuador; to identify trade-offs between these considerations; and to understand how an evolving urban environment influences these trade-offs.

**Methods:**

Semistructured interviews were conducted with 20 mothers of young children to explore drivers in food choice throughout the purchase, preparation, and consumption chain. Interviews were transcribed and coded to identify key themes.

**Results:**

Personal preference, economic access (costs), convenience, and perceptions of food safety were key influencers of decision making related to food. In addition, concerns about personal safety in the urban environment limited physical access to food. This, combined with the need to travel long distances to obtain desirable foods, increased men’s participation in food purchasing. Women’s increasing engagement in the workforce also increased men’s participation in food preparation.

**Conclusions:**

Policies to promote healthy food behavior in this context should focus on increasing access to health foods, such as affordable fresh produce, in convenient and physically safe locations. *CurrDev Nutr* 2023;x:xx.

## Introduction

Worldwide, shifts from traditional to diets that are high in fats and refined carbohydrates have been followed by increases in overweight and obesity [1,2]. This nutrition transition is of concern because the elevated consumption of low-quality refined carbohydrates, saturated fats, and sodium are among the leading causes of morbidity worldwide [3,4]. In Latin America, as in other parts of the world, changing dietary patterns have been linked to ongoing food system transformations [2], resulting in calls to better support food environments that promote healthy diets [5]. The food environment refers to the assortment of foods that are available to individuals in the physical spaces they occupy as they go about their daily lives [5,6]. These environments are characterized by both “external” domains such as food availability, food cost, characteristics of the food vendor and/or the food product, and marketing and regulation as well as “personal” domains such as accessibility, affordability, convenience, and desirability [7,8].

Food environments are not static and evolve over time in response to broader economic, political, and social forces, including increasing global urbanization [9]. Urban areas possess large markets, which support large-scale food retailers and options for specialized foods [10]. These factors have the potential to sustain nutritious diets through the increased availability of diverse foods [5]. However, other characteristics of urbanization may counteract this benefit. Sedentary lifestyles, increased exposure to marketing and the wider availability of energy-dense, nutrient-poor foods, may contribute to the nutritional transition for urban dwellers, especially those living in cities where the built environment does not promote physical activity [11], or food advertising and sales are under-regulated [12] This is particularly true in Latin America, where advertising and food pricing may promote the sale of processed compared with fresh foods [13].

Although the food systems of the largest urban areas have often been the best characterized, there are growing calls to better understand intermediate-sized cities (generally with a population of 100,000–500,000), also called secondary cities [14]. The residents of secondary cities are often of relatively low socioeconomic status, making them vulnerable to food insecurity [15], and often face multiple burdens of child undernutrition, micronutrient deficiencies, and overweight and obesity [16]. Secondary cities have similarities and differences to primary urban centers. Because their food systems sit at the boundary between urban and rural means of production, distribution, and consumption [17], they are influenced by both the nearby agricultural sector and global markets [18]. For instance, families living in these cities often source food from rural connections [19,20], and may be impacted if smallholder farms are replaced by larger-scale agriculture [21]. Secondary cities are also growing rapidly because of rural-to-urban migration, which, in turn, has resulted in a greater presence of larger food distributors [22].

Food environments in Ecuador are diverse and include markets, neighborhood stores, supermarkets, mobile food vendors, and both formal and informal prepared-food establishments. Traditional markets, which are set up and administered by local municipalities, are highly used [23]. These markets are a common final point of sale for consumers as well as for market vendors and neighborhood stores [[23], [24], [25]]. Supermarket chains, which first emerged in the 1990s, continue to rapidly expand their influence [26,27]. The supermarket sector is dominated by a few national and multinational companies, which have strengthened their reach by creating different store formats to cater to consumers across the socioeconomic and urbanization spectrums, including those of lower socioeconomic levels and those living in smaller cities [22,27]. These strategies have led to the targeted expansion out of the major cities of Quito and Guayaquil into developing areas, including the coastal city of Esmeraldas [28].

Although Ecuador has historically implemented nutrition policies and programs aimed at addressing undernutrition in children [29], the prevalence of obesity has increased rapidly in the past decade, now estimated at 44.2% among adults [30,31]. Although the burden of obesity remains greatest in the largest urban centers, obesity is also a growing concern in secondary cities and the rural areas that surround them [30,31]. In response, Ecuador enacted the 2014 traffic light food labeling system as a public policy to inform and warn consumers via labels of foods with excess amounts of fats, sugar, or sodium [32]. In 2020, food-based dietary guidelines were released for the country [33]. The food guide is represented by a wooden spoon and includes 11 key recommendations that are meant to reflect variety, proportionality, and cultural diversity [33]. An additional program, 2013 Healthy Food Market Program, calls for spaces that are clean, hygienic, safe, and promote local traditions and customs [34]. Compared with the Healthy Food Market Program and the newer dietary guidelines, the traffic light labeling has been extensively evaluated by both government and academic groups. Evidence shows that the labeling system is easily recognized and understood but is not often used by consumers and has not had impacts on purchasing patterns [[35], [36], [37]].

Recognizing the importance of changing food environments in secondary cities, we conducted semistructured interviews in the coastal Ecuadorian city of Esmeraldas. Our aims were to: *1*) describe considerations that drive household food purchasing, preparation, and consumption behavior among women in Esmeraldas, Ecuador; *2*) to describe trade-offs between these considerations; and *3*) to understand how characteristics of the urban environment might influence these considerations.

## Methods

### Study context

The study was conducted in the city of Esmeraldas, located on the northwestern coast of Ecuador. Esmeraldas province has high rates of poverty because of unmet basic needs, exceeding the national average and ranking fifth among the country’s 24 provinces [38]. Esmeraldas City is the capital of Esmeraldas province. The city has a population of 154,000 and is home to the second highest urban Afro-Ecuadorian population in the country [39,40].

**FIGURE 1:**
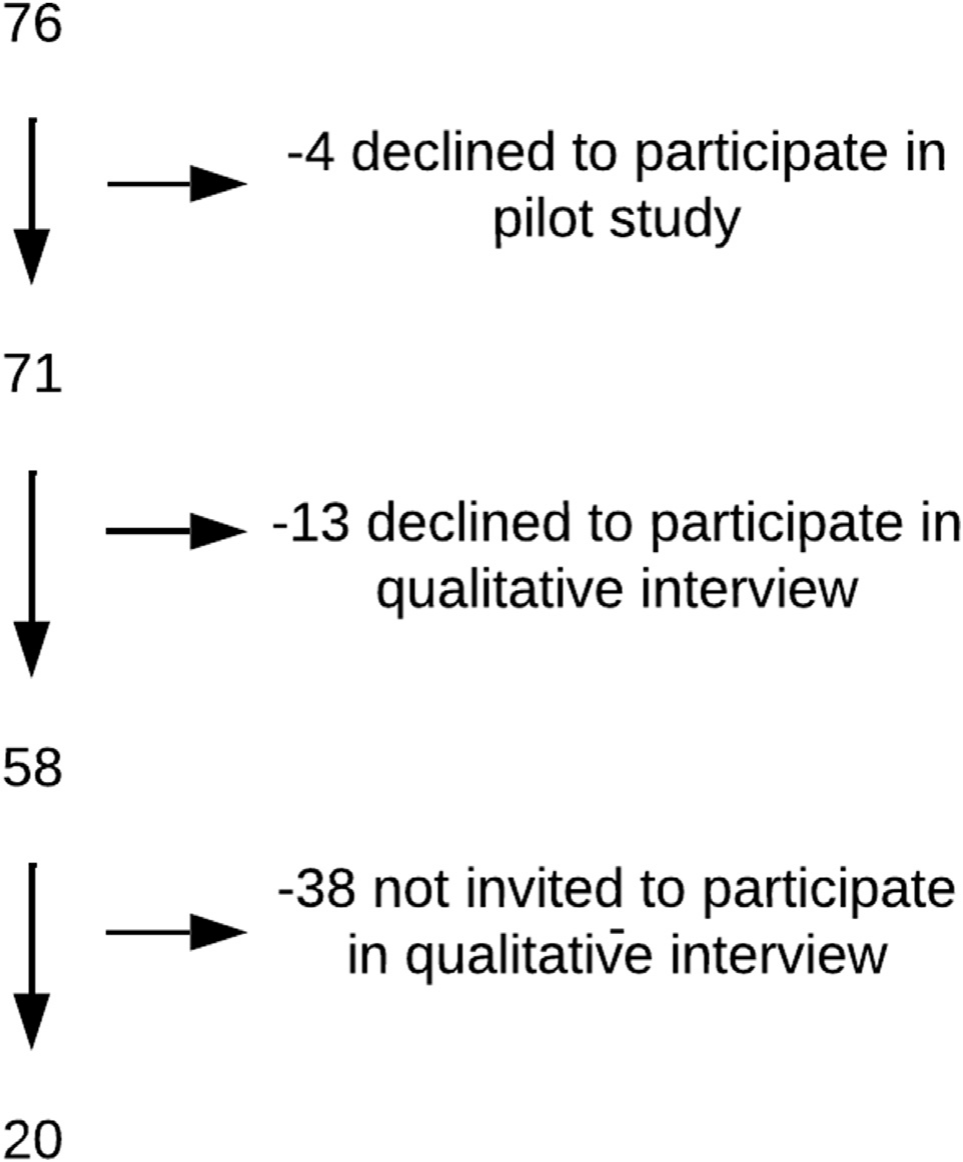
Study CONSORT flow diagram.

At the time of the study, the city of Esmeraldas had 7 supermarkets, making it comparable with other Ecuadorian cities of similar size, such as Quevedo and Ambato [[41], [42], [43]]. All 7 establishments are owned by 2 multinational companies operating the largest supermarket chains in the country: Tiendas Industriales Asociadas S.A. with over 200 locations, and Corporación Favorita C.A. with over 140 locations [28,41,44]. Five of the supermarkets were clustered within the city center and 2 were located adjacent to the provincial bus terminal at the city’s southern end (Figure 1) [45]. Located in the city center, the Esmeraldas municipal market, built over 30 y ago, is the only formal market servicing the city [45]. The market has 500 vendor capacity and has fruits, vegetables, meat, seafood, and dry goods available for purchase [46]. Informal and formal food vendors can also be found in the area surrounding the market. Throughout the city, neighborhood stores, which account for 75% of all national food and drink sales [47], are common. At the northeastern side of the city, the Esmeraldas fishing port, inaugurated in 2016, is a source of fresh seafood [48].

### Sampling

We conducted qualitative interviews with 20 mothers of young children. These interviews were nested within a cross-sectional pilot survey of 71 mother–child dyads. The primary objective of the cross-sectional pilot survey was to refine study instruments and protocols for the “Gut microbiome, enteric infections and child growth across a rural–urban gradient” birth cohort study [49]. The primary, a priori objective of the qualitative substudy was to better understand how urbanicity might drive household decision making around food for families with young children. Participants for the pilot survey study were identified using a combination of recruitment at a district health center and snowball sampling (referral by other participants). Inclusion criteria included being a mother of age 18 y or older who self-identified as Afro-Ecuadorian and had ≥1 child 2 y of age or younger with no known major health issues. At the beginning of enrollment for the pilot survey, mothers who provided written informed consent to participate were additionally asked if they were willing to participate in an audio-recorded qualitative interview. After the study team decided that their sample size for the qualitative interviews was sufficient because information saturation had been reached, later enrollees to the pilot survey were not invited to participate in the additional interview (Figure 1).

### Data collection

Data collection took place from June to August 2018. Before the interviews, 4 trained nurse technicians or nurse auxiliaries, who had previously received a 1-wk training in research ethics and specific study aims and instruments, administered questionnaires to collect participant demographics and information pertaining to food purchasing and preparation behavior. Demographic information included ethnicity, age, birthplace, occupation, marital status, and parity. Food purchasing questions were derived from Ecuador’s 2010 Socioeconomic Level Stratification survey [50].

Semistructured interviews were used because of their flexibility for drawing on interviewee answers to guide conversation and explore new ideas that may be relevant to understand local drivers of food choice [51]. An interview guide was developed to explore decisions and behavior guiding each stage of the food purchasing, preparation, and consumption chain as well as knowledge about nutrition and processed foods. Questions were reviewed by field staff for appropriateness and comprehensibility. Participants were interviewed in their home by a native Spanish speaker (JU) and conversations were recorded using a portable audio recorder. Members of the study team (JU and GOL) met throughout the data collection to discuss the findings. Interviews were conducted until thematic saturation was reached, that is, until no new patterns or themes are emerging from the data [52].

### Human subjects

Study protocols and instruments were approved by the ethical review boards at the University of Michigan and the Universidad San Francisco de Quito. Mothers participating in the pilot survey received a food basket of value USD3 as a gift of appreciation for their time participating in the survey, and mothers participating in the qualitative interviews received an additional USD3 gift of an infant t-shirt. To protect the anonymity of the subjects, all data were collected in a deidentified manner, using study ID codes to identify participants in all study documentation other than the consent forms themselves. The consent forms were stored by the study staff in a secure cabinet in the study team’s office, which was located within the public Hospital Delfina Torres. When data collection ended, all consent forms were scanned, the scans were stored in a private cloud storage service managed by the University of Michigan, and the original article consent forms were shredded. Any names mentioned during audio interviews were excluded from transcripts, and, after transcriptions were complete, the original audio was destroyed. Because the analysis was not completed in real time, we were unable to inform participants of the results of the study.

### Analysis

Calculations of means, SDs, and tabulations of percentages of questionnaire data were completed using R (version 4.0.3, R Core Team). To visualize the study area, a map was created in QGIS Software [53] using publicly available geographic data from Google Maps and a Snazzy Maps style licensed under the creative commons [54]. Audio recordings of the interviews were transcribed in Spanish by an Ecuadorian transcriptionist. Transcriptions were coded and analyzed by 2 coders, 1 of which was the interviewer. All interviews were analyzed in Spanish, without translation. Interview coding was completed using Dedoose (version 8.3.45, SocioCultural Research Consultants LLC). We used a content analysis approach [55]. Initial structural codes were based on themes covered by the interview guide, as well as key dimensions of low- and middle-income country (LMIC) food environments reported in the literature, as summarized by Turner et al. [8]. These included accessibility, affordability, convenience, and desirability, among others. We also coded the activity described (for example, purchase, preparation, or consumption), the people who participated in the activity (for example, infants or young children), the kinds of food mentioned (for example, homemade or restaurant food), and directionality in relation to the kinds of food mentioned (for example, healthy or unhealthy, inexpensive or expensive). After a careful reading of the data by each researcher, additional codes were added on the basis of topics that emerged unexpectedly from the data, primarily dimensions of the food environment that had not been included a priori. The researchers met weekly to discuss code definitions and decide on the final coding. After both researchers had separately coded all interviews, results were compared with assess reliability. Finally, to visualize the data, we counted the number of instances in which key codes related to domains of the food environment occurred in combination with codes for food purchase, preparation, and consumption [55].

## Results

### Survey results

**FIGURE 2:**
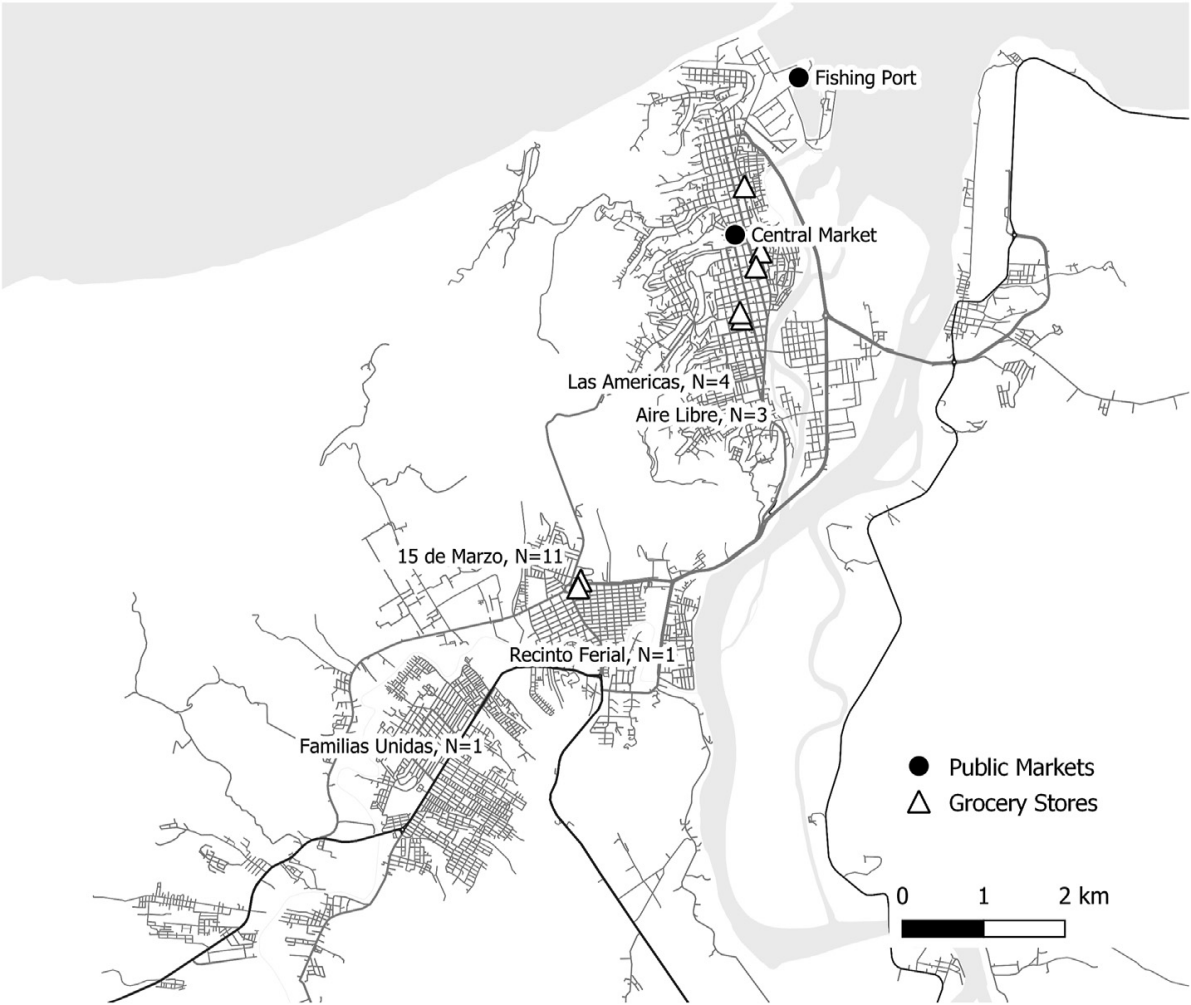
Food outlets and participant neighborhood at the time of the study.

Twenty women participated in this study. These women lived in 5 neighborhoods of the city of Esmeraldas. Most (13/20) lived in neighborhoods located in the southern part of the city (Table 1). These neighborhoods are more “suburban” in character and are close to the 2 supermarkets located at the provincial bus terminal but relatively far from the municipal market (∼30 min by public transportation). The remaining women lived in neighborhoods that were closer to the city center and thus are more “urban” in character, as well as proximal to the municipal market and the other supermarket locations (Figure 2).

**TABLE 1:** Participant demographic information

Variable	*N* = 20^1^
Neighborhood	
15 de Marzo	11/20 (55%)
Aire Libre	3/20 (15%)
Familias Unidas	1/20 (5%)
Las Américas	4/20 (20%)
Recinto Feria	1/20 (5%)
Ethnicity	
Afro-Ecuadorian	19/20 (95%)
Other	1/20 (5%)
Age	24.0 (21.0, 29.5)
Birthplace	
Esmeraldas City	16/19 (84%)
Other	3/19 (16%)
Main occupation	
Homemaker	11/19 (58%)
Student	7/19 (37%)
Teacher	1/19 (5.3%)
Lives with their child’s father	15/19 (79%)
Marital status	
Married or in a couple	13/19 (68%)
Never married or never in a couple	6/19 (32%)
Age at first pregnancy	18.0 (17.0, 24.0)
Number of children	
1	7/19 (37%)
2	6/19 (32%)
3	6/19 (32%)

Birthplace, occupation, marital status, and number of children were missing for 1 respondent (total of 19 rather than 20). ^1^*n*/*N* (%); median (IQR).

Women reported an average monthly food expenditure of USD150. Most women selected neighborhood stores as the location they most frequented when purchasing food, followed by markets, and lastly supermarkets. Around a third (37%; 7/19) of women reported shopping for groceries alone and 84% (16/19) reported preparing meals 3 times/d (Table 2).

**TABLE 2:** Participant food purchasing and preparation behavior

Variable	*N* = 20^1^
Monthly food expenditure (United States dollars)	150 (120, 224)
Most frequented food purchasing location	
Neighborhood store	11/19 (58%)
Markets and fairs	7/19 (37%)
Other	1/19 (5.3%)
Shops for groceries alone	7/19 (58%)
Prepares a shopping list	
No	13/19 (68%)
Sometimes	4/19 (21%)
Always	2/19 (11%)
Shared cooking responsibility	11/19 (58%)
Household daily food preparation frequency	
1 time	1/19 (5%)
2 times	1/19 (5%)
3 times	16/19 (85%)
Other	1/19 (5%)

1 *n*/*N* (%); median (IQR).

### Interview results

Semistructured interviews ranged in length between 15 and 50 min (mean of 28 min). Interviews discussed key dimensions of the food environment impacting food purchasing, preparation, and consumption decisions, and trade-offs between them. The interview guide is provided in the Supplemental Material.

#### Key dimensions of the food environment and trade-offs between them

**FIGURE 3:**
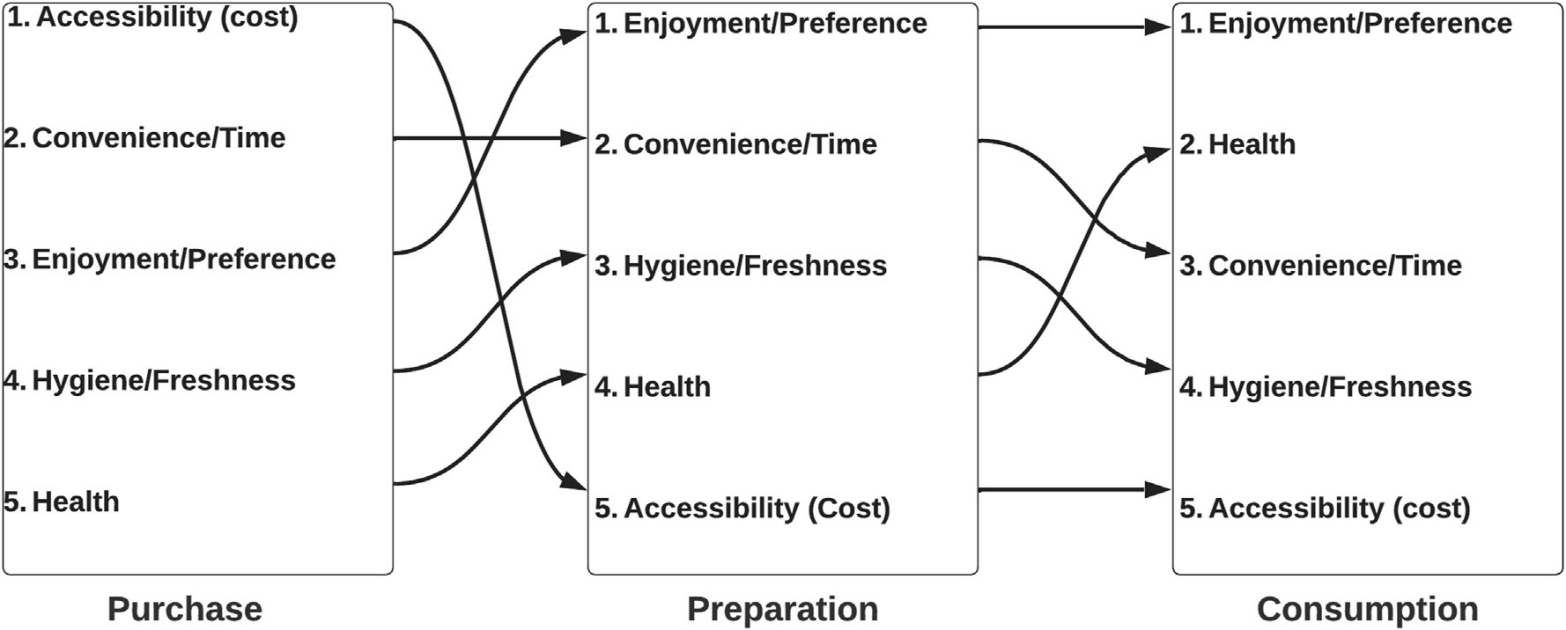
Factors influencing food choices, ranked by the frequency with which they were discussed in semistructured interviews.

From the interviews, the most frequently discussed dimensions of the food environment were economic and physical accessibility (cost and distance), convenience (time), hygiene and freshness, health, and enjoyment/preference. The relative importance of these dimensions was ranked by the opinion of the interviewees and visualized according to the frequency with which they were discussed in relation to food purchase, preparation, and consumption (Figure 3). Participants also often discussed trade-offs between these dimensions when making decisions around food.

##### Purchasing

Economic accessibility was the most influential driver of purchasing decisions. Cost was frequently discussed as the deciding factor of where to acquire food, as well as what to purchase.

When describing what foods to buy, participants often discussed their purchases hierarchically based on cost, “building toward” a complete, traditional Ecuadorian meal that consists of soup, rice and beans, protein (eggs, fish, poultry, or meat), and fruit juice. Staples, such as rice, beans, oil, and eggs, were prioritized first. Households with the economic means to do so supplemented these with chicken, seafood, or red meat, followed by vegetables and fruits last. Other considerations were typically outweighed by concerns over cost.

After cost, physical access—described in terms of convenience relative to travel time and concerns over personal safety—also influenced purchasing decisions. The municipal market was generally viewed as having the lowest prices. However, long travel times were a barrier to visiting the municipal market, as were concerns about robbery. Nevertheless, a preference for lower cost and higher quality fresh foods also meant that almost all families still shopped at the municipal market on a weekly, biweekly, or monthly basis. Given the relative inaccessibility of the municipal market, participants also relied on neighborhood stores to supplement these large trips. Multiple participants mentioned that concerns over personal safety had led their husbands to take a more active role in food purchasing. For instance, 1 woman explained that she only went to the market if she was accompanied by her husband because of past experiences of being robbed:

*“…sometimes when I’ve gone to the market it’s gone badly for me because I always get robbed, haha, it’s a little dangerous plus I am fearful that’s why, if I go with my husband, on a Sunday when he’s here then I go to the market if he accompanies me, but I don’t go to the market alone.” [20-y-old woman]*

In other households, safety concerns or concerns related to cost and ease of transportation led husbands to handle most larger food purchases alone. Some women said that they provided their husbands with lists of what to purchase, whereas in other instances, the husband had “learned already” what to buy.

Food freshness was also an important consideration during food purchasing, especially for young children at risk of diarrheal disease. Visual and tactile cues were important indicators of freshness that influenced where food items were bought. Supermarket meats were not perceived as fresh because of their dull color, dryness, and long refrigeration times. On the other hand, municipal market meats were considered fresh because of their daily preparation and vibrant color. A preference for freshness also led most participants to prefer municipal market fruits and vegetables over grocery store produce and caused some participants to buy food from neighborhood stores daily, despite often having higher prices. Brand and store preferences were also described relative to food hygiene concerns: 1 participant believed that nonpasteurized milk contained added water, whereas another participant mentioned a belief that some food establishments added formaldehyde to their poultry, leading them to opt for some stores while foregoing others.

In addition to health concerns related to risk of diseases from unhygienic or chemically contaminated foods, the perceived healthiness of foods was also considered in relation to purchasing decisions. However, as 1 participant explained, what is considered a healthy diet (fruits and vegetables) was oftentimes unattainable given their economic circumstances:

*“As I told you, fruits. Vegetables, […] but sometimes, for example, the poor can’t follow a strict diet like that, you understand me, because as a poor person you have to eat what there is, if today you have enough to make beans with rice which are both carbohydrates, that’s what you have to eat, you understand me.” [35-y-old woman]*

##### Preparation and consumption

All participants cooked daily for their family. Family preferences emerged as the most significant driver of decision making related to food preparation. Familial preference was often described alongside—or in contrast with—concerns over convenience or time management. For instance, adhering to overall family preferences was discussed as a strategy to avoid preparing multiple dishes for different family members. However, it was possible for preference to overtake convenience, especially for young children, as illustrated by a participant who accounted for the varying preferences of their family members by preparing multiple dishes for the same mealtime.

*“when I make shrimp, I know that I have to give the child rice with egg, or rice with tuna, 1 of those 2, because he doesn’t eat shrimp no matter how many times I try, he won’t eat it, he doesn’t like it” [35-y-old woman]*

Similar to discussions around cost, convenience was often described by comparing what a participant actually prepared with a typical “complete, traditional” Ecuadorian meal of soup, rice, beans, animal-source protein, and juice. For instance, 1 participant did not cook soups, whereas another participant opted to prepare either soup or rice at mealtimes, but not both. Decisions to cook less than a complete traditional Ecuadorian meal were often described as a way to fulfill cooking responsibilities while accommodating other household obligations.

Food hygiene was also raised as an important consideration during meal preparation. Participants washed ingredients with lime juice and salt to remove the slimy texture from the surface of their food and clean food that may have been exposed to insects. Chicken, pork, beef, and seafood were all washed in this manner. One participant mentioned sometimes also using soap to wash their food, whereas another rinsed their chicken with hot water after being shown the procedure by a person they identified as a nutritionist. The participant was so greatly impacted by this experience that they began to avoid consuming chicken, even when prepared by their family, unless they were able to confirm that it was washed with hot water.

.*”.in general I wash it with water, I wash it with a lot of salt and lime, I leave it to cure for a while from there I wash it, but with chicken, if I do not pour hot water I do not eat it, even if my sisters cooked it I don’t eat it… I ask did you add hot water she tells me yes then yes, but if she tells me no, no I did not add it, I do not eat it, aha, because of that I do not like it after seeing that, because the water looked very dirty, very.” [34-y-old woman]*

Other considerations, such as health, were also often noted in the context of preference, convenience, or food safety. For instance, 1 participant noted that lentils were the most frequently prepared dish not only because it was the most economical choice but also because they believed it had high vitamin content. Health concerns related to potentially contaminated foods were expressed as or more often than concerns related to the nutritional value of the foods consumed. Although most participants were aware of the traffic light food labels, no participant described using the traffic light food labels to help them make food choices. For example, 1 participant noted:

*“Right now, these labels about high salt and things like that have appeared; however, I eat whatever and I don't read anything. I go and eat, and eat, and I eat in quantity, that is to say a lot, I say it must be bad I say”*

*[Interviewer] “So the labels, you’re telling me that you pay attention to them or.?”*

*“When I finish eating, haha” [20-y-old woman]*

Decisions related to food consumption were closely related to decisions around food preparation, with some exceptions. In contrast to food preparation, which most women managed alone, consumption was most often described in terms of eating as a family, with an emphasis on familial enjoyment and traditional gender roles (the woman serving her husband and children). Again, other considerations, such as cost, were often noted in the context of preference—many women preferred to serve food to their families that they knew were preferred and enjoyed, to avoid food waste. Although most participants emphasized the importance of the traditional family meal, served by the mother and eaten together, women who were working or studying outside the home also described an increased reliance on convenience foods that could be eaten quickly or on the go.

Consumption of some foods was avoided altogether because of food hygiene concerns. One participant had stopped eating liver after hearing bacteria and other “bad things” from the cow accumulated there. Four participants reported that they entirely avoided foods prepared outside the home, whereas others specified that they ate out on special occasions. In 1 case, a social media post describing unclean commercial food preparation areas led a participant to prefer cooking the foods they craved, even if it did not turn out well, instead of going out to eat. In another case, a participant expressed their frustration with prepared-food vendors who they believed do not care for the health of their customers.

*“It’s that sometimes people, to earn money, don’t care how they prepare those things, they don’t care if the water is dirty, if it’s clean and all that, that is, they don’t look at the sugar because many times those little ants tend to get in the sugar…they are not fully complying because they save today’s oil for tomorrow supposedly to save money because they do not care about the health of others and in general they are mostly children” [32-y-old woman]*

Several participants also mentioned an aversion to storing food for later consumption because of food hygiene concerns. For this reason, they prepared just enough food for their family to avoid leftovers. When food was left over, most participants preferred to use it for other meals within the same day, whereas a few would store leftovers for the next day and consume it only after inspecting it to determine that it had not spoiled. One participant explained that leftovers were consumed by the adults in the family, but not by the children because it was bad for them.

*“What do I do with leftovers is that I store but for the 2 of us who are the oldest, but for the children no because there are foods that can hurt children from 1day to the next, so for him nothing from 1 day to the next, everything has to be fresh.” [24-y-old-woman]*

## Discussion

As in many urban food environments globally [[56], [57], [58]], external drivers including physical and economic access are interconnected factors that influence food choice in the city of Esmeraldas in Ecuador. At the same time, internal drivers such as traditional gender roles, personal and familial preferences, and a strong desire for fresh, safe foods, maintain a food culture dominated by traditional markets and home cooking [17,57]. Although many of these factors can help to support healthy diets, others may negatively impact dietary patterns for this population.

There were some factors that may have been more relevant to Esmeraldas compared with other LMIC settings. Specifically, significant concerns over robbery or other threats to personal safety reduced physical access to some food sources. Unfortunately, the frequent robberies reported in the city of Esmeraldas at the time of the study further increased during the COVID-19 pandemic and became severe as feuding organized criminal organizations caused a state of emergency to be declared in 2022 [59,60]. This threat of violence—alongside other factors such as the growing role of women outside the home—resulted in increased male participation in food purchasing.

Food safety, a topic that came up as important in our study, is also a domain that influences food-related decision making in many LMIC settings [[61], [62], [63]]. Several authors have now reported that food safety concerns may lead consumers to purchase and consume more ultraprocessed foods and fewer fresh foods [64,65]. In our study, concerns around food safety—phrased in terms of food freshness and hygiene—were most often referred to in the context of fruits, fresh vegetables, dairy, and meat. The preferred source for produce and meat was the municipal food market, which sold the products that families felt were freshest and safest. However, financial constraints led families to purchase these foods only after basic staples, such as rice, oil, and beans, were obtained. The relatively long travel times and lack of physical safety in the municipal market may have reduced the purchase of these foods. Relatively few studies have linked perceived food safety concerns to microbiological and chemical contamination [61]. However, produce, dairy products, and meat are often at a relatively high risk of contamination [66]. These same foods are key to maintaining a nutritious diet. Although programs such as the Healthy Markets initiative (described in the introduction) are well positioned to address food safety concerns by supporting hygienic traditional food markets [34], there is also a need to ensure the physical and economic accessibility of these spaces.

Creating effective interventions to improve diets requires an understanding of barriers to healthy food choice. We found that the “traffic light” food labels used in Ecuador had little if any effect in determining food purchasing and consumption decisions, which has been shown in several studies both nationally [32,[67], [68], [69], [70], [71], [72]] and internationally [73,74]. Although participants were aware of labels, food prices, personal preference, and convenience held a greater influence on decision making than health. In addition, food labels that emphasize sugar, fat, and salt intake may be imperfectly aligned with concerns about health that are oriented as much toward avoiding microbiological contamination and diarrheal disease as toward reducing chronic disease risk. Again, pairing labeling with improvements in economic and physical access to healthy and safe foods may increase the ability of consumers in Esmeraldas to prioritize nutritional value highly within their food choices.

Our exploratory qualitative study had limitations. First, recruitment of participants occurred through snowball sampling and does not provide a representative sample. Although participant neighborhoods covered a wide geographical region of the city, not all socioeconomic levels were proportionally represented, which may limit the generalizability of the results. Geography may have affected participants’ visits to the municipal market, which were relatively far from the “15 de Marzo” neighborhood where many of our study participants lived, and to supermarkets, which were correspondingly close by. Despite this, almost all participants preferred the municipal market to the supermarket. Second, although this study focused only on women with young children, our results suggest that men play a growing role in food purchasing. However, the perspective of men was not directly captured, given that our interviews only occurred with women. Finally, it is possible that responses were influenced by participant attempts to conceal personal family situations and provide socially desirable responses. However, this work provides a starting point from which future research can continue to define the nuances that contribute to food choice behavior, both in Esmeraldas and in other LMIC secondary cities.

Although preferences for diverse, fresh, homemade foods align with Ecuadorian dietary recommendations [33], the extent to which urban families in Esmeraldas can carry out these recommendations is constrained by cost, a challenging urban environment, time constraints, and food safety concerns. Increasing male participation in food purchasing implies that intervention strategies should target both men and women. On a structural level, efforts to address urban violence and poverty in Esmeraldas would indirectly support healthy diets by promoting physical and economic access to affordable foods. More targeted nutrition policies can leverage existing preferences by combining messages around health with messaging around food safety, the promotion of convenient and healthy choices for busy families, and by emphasizing affordable traditional markets that are physically accessible and safe and resonate with existing perceptions of fresh and healthy foods.

## Author contributions

The authors’ responsibilities were as follows—JU and GOL: designed the research; JU: conducted the research; WC, NC, KL, and JNSE: provided essential materials; JU and GOL: analyzed the data; JU, CR, and GOL: wrote the article; KL, JNSE, and GOL: had primary responsibility for the final content; and all authors: read and approved the final manuscript.

## Data availability

Data described in the manuscript, code book, and analytic code will be made available upon reasonable request pending approval by the Universidad San Francisco de Quito ethical review board.

## Funding

This work was supported by the United States National Institutes of Health (grant numbers R01AI137679; K01AI145080). JU was supported by the University of Michigan Office of Global Public Health at the School of Public Health and the University of Michigan International Institute. The content is solely the responsibility of the authors and does not necessarily represent the official views of the National Institutes of Health. The funders had no role in the design, implementation, analysis, or interpretation of the data.

## Author disclosures

JU, CR, NC, WC, KL, JNSE, and GOL, no conflicts of interest.
