# Supplementary material for: A Qualitative Study of Food Choice in Urban Coastal Esmeraldas, Ecuador
Source: Curr Dev Nutr. 2023 Apr 26;7(5):100093. doi: 10.1016/j.cdnut.2023.100093 (PMC10206432; doi:10.1016/j.cdnut.2023.100093)
Supplement: Multimedia component 1 [file mmc1.docx]

**Supplemental Materials**

**Interview Guide (English)**

Today we will be talking about the issues of buying, preparing, and eating food. During our conversation I will ask you several questions, which do not have a right or wrong answer. I would simply like to learn about your experiences and opinions regarding these issues to have a better understanding of the factors that influence food consumption in this community. If you have any questions during our conversation or don't understand one of the questions I'm asking, feel free to let me know. I have a voice recorder with me that I will use to record our conversation to ensure that all the information that you have taken the time to give me is captured. At this time the recorder is turned off. Do you have any questions before we start? [Answer any questions the participant has.]

Okay, now I'll turn on the recorder and we'll start our conversation.

**Introduction**

Before I start with the questions I have, I think it would be a good idea to introduce ourselves to each other to get to know each other a little better and thus make our conversation more comfortable. It looks good? [Interviewer introduces himself] Now, could you tell me a little about yourself?

**Food Purchase**

There are different types of food such as fruits, vegetables, grains, fish, meat, packaged goods, drinks, and the list goes on. How do you decide what type of food you will buy or consume? [Probes: When you have to buy food, what do you think about when you make the decision of what produce or packaged products to buy?

Now I would like to talk about where you get your food from. Can you tell me what kind of places you visit to get food and what are the reasons why you visit those particular places? [Probes: Where do you go to get food? Why do you visit this place instead of another place that also sells the products you need?]

Sometimes food prices can go up or down. Can you tell me if you have noticed or have not noticed that any food has changed in price recently?

There are also sometimes when some food is available or not available to buy. Can you tell me about the availability of food throughout the year?

**Food preparation**

What food do you cook most often? Why is the kitchen eaten more often?

What food do you cook less often? Why is the kitchen eaten less often?

There are certain things that can make the task of cooking easy or difficult. What, if anything, does exist that makes the task of cooking easy for you?

What, if anything, does exist that makes the task of cooking difficult for you?

**Food Consumption**

At mealtimes, some families eat together while others do not. Can you tell me about your family's mealtime routine? [Probes: Does the whole family gather at mealtimes or just some family members? How is the food served, do you serve everyone by yourself, does someone help you, or does each family member serve himself?]

**Knowledge of Processed Food**

These days people talk a lot about nutrition and healthy food. What meaning do these terms have to you and what do you know about them? [Probes: When you hear the word nutrition, what do you think of? What are some things you have heard about healthy eating?]

Have you heard of processed food? What do you know about this type of food? [Probes: What is processed food to you? What do you think is good or bad about processed food?]

**Guía de Entrevista (Español)**

El día de hoy estaremos hablando acerca de los temas de la compra, preparación, y consumo de la comida. Durante nuestra conversación le hare varias preguntas, las cuales no tienen una respuesta correcta o incorrecta. Simplemente me gustaría aprender acera de sus experiencias y opiniones con relación a estos temas para tener un mejor entendimiento sobre los factores que influyen en el consumo de la comida en esta comunidad. Si usted tiene cualquier pregunta durante nuestra conversación o no entiende una de las preguntas que le hago, no dude en dejarme saber. Tengo conmigo una grabadora de voz que utilizaré para grabar nuestra conversación y de ese modo asegurarme que toda la información que usted ha tomado su tiempo para darme sea captada. En este momento la grabadora se encuentra apagada. ¿Tiene usted alguna pregunta antes de comenzar? *[Responda cualquier pregunta que la participante tenga.]*

Bien, ahora prenderé la grabadora y comenzaremos nuestra conversación.

**Introducción**

Antes de comenzar con las preguntas que tengo, pienso que sería una buena idea presentarnos la una a la otra para conocernos un poquito mejor y para que de ese modo nuestra conversación sea más cómoda. ¿Le parece bien? *[El entrevistador se presenta a si mismo]* ¿Ahora, me podría contar un poco sobre usted?

**Compra de Comida**

Existe diferentes tipos de comida por ejemplo frutas, vegetales, granos, pescado, carne, productos envasados, bebidas, y la lista continua. ¿Cómo usted decide qué tipo de comida usted comprará o consumirá? *[Sondeos: ¿Cuándo tiene que comprar comida, en que es lo que piensa cuando hace la decisión de que productos agrícolas o envasados va a comprar?*

Ahora me gustaría hablar sobre los lugares de donde usted consigue su comida. ¿Me puede decir que tipo de lugares usted visita para conseguir comida y cuáles son las razones por la cual usted visita esos lugares en particular? *[Sondeos: ¿Dónde usted va para conseguir comida? ¿Por qué visita este lugar envés de otro lugar que también vende los productos que usted necesita?]*

Algunas veces los precios de la comida pueden subir o bajar. ¿Me puede contar si usted se a dado cuenta o si no se ha dado cuenta de que alguna comida ha cambiado de precio recientemente?

También hay algunas veces cuando alguna comida está disponible o no está disponible para comprar. ¿Me puede contar sobre le disponibilidad de la comida durante el año?

**Preparación de Comida**

¿Qué comida cocina usted con más frecuencia? ¿Por qué cocina está comida con más frecuencia?

¿Qué comida cocina usted con menos frecuencia? ¿Por qué cocina está comida con menos frecuencia?

Hay ciertas cosas que pueden hacer la tarea de cocinar fácil o difícil. ¿Qué, si algo es que existe, hace la tarea de cocinar fácil para usted?

¿Qué, si algo es que existe, hace la tarea de cocinar difícil para usted?

**Consumo de Comida**

A la hora de comer, algunas familias comen juntas mientras que otras no lo hacen. ¿Me puede contar sobre la rutina que su familia tiene a la hora de comer? *[Sondeos: ¿A la hora de comer se reúne toda la familia o solo algunos miembros de la familia? ¿Cómo se sirve la comida, usted sola sirve la comida para todos, alguien la ayuda, o cada miembro de la familia se sirve solo?]*

**Conocimientos de la Comida Procesada**

Estos días las personas hablan mucho de la nutrición y la comida saludable. ¿Qué significado tienen estos términos para usted y que es lo que usted conoce acera de ellos? *[Sondeos: ¿Cuándo escucha la palabra nutrición, en que es lo que usted piensa? ¿Qué son algunas cosas que usted ha escuchado sobre la comida saludable?]*

¿Ha usted escuchado de la comida procesada? ¿Qué es lo que usted conoce acera de este tipo de comida? *[Sondeo: ¿Qué es la comida procesada para usted? ¿Qué es lo que piensa que es bueno o malo de la comida procesada?]*
